# Adapting to low phosphorus: Alterations in *HvSPX4* expression levels in barley

**DOI:** 10.1093/plphys/kiaf443

**Published:** 2025-09-26

**Authors:** Munkhtsetseg Tsednee

**Affiliations:** Assistant Features Editor, Plant Physiology, American Society of Plant Biologists; Research Center for Environmental Changes, Academia Sinica, Taipei 11529, Taiwan

The crop productivity of cereals, such as wheat and barley, relies largely on phosphorus (P) fertilizer. This is particularly common for soils with low or high pH, where bioavailable inorganic P is limited due to its conversion to organic forms (Yang et al. 2024). Yet, some genotypes within crop species exhibit tolerance to low P stress—for example, Dular and Kasalath in rice (Mehra et al. 2015); landrace in sorghum (Leiser et al. 2014); and GN121, X130, and Zaoaibai in barley (Ren et al. 2018; Zhang et al. 2024). Understanding the molecular mechanisms underlying low P tolerance in these genotypes is crucial for developing crops with enhanced P mining and utilization efficiency.

In a recent issue of *Plant Physiology*, Zhang et al. (2025) investigated P regulations in the low P–tolerant barley genotype Zaoaibai and identified the critical function of a regulator gene, *HvSPX4*, for its tolerance under low P. *HvSPX4* is a member of the *SPX* (*SYG1/Pho81/XPR1*) gene family. It acts as a negative regulator in P deficiency response by interacting with key players—namely, phosphate starvation response (PHR) proteins (Rubio et al. 2001)—in Arabidopsis (Osorio et al. 2019) and rice (Lv et al. 2014). However, the *SPX4* function in barley remains unexplored.

In a previous transcriptome analysis of barley genotypes (Zhang et al. 2024), the authors observed lower expression of *HvSPX4* in the low P–tolerant genotype Zaoaibai than the low P–sensitive genotype Salooni2. In this study, they confirmed that regardless of P supply, *HvSPX4* mRNA accumulation in Zaoaibai tissues was reduced when compared with Salooni2. The phylogenetic analysis also showed that HvSPX4, out of 12 SPX domain–containing proteins identified in barley, has high similarity to OsSPX4 and AtSPX4, which prompted the authors to examine its role in low P response.

The mutation of *HvSPX4*, as seen in their CRISPR/Cas9 knockout lines, further resulted in reduced biomass and increased P accumulation under sufficient P conditions, whereas no obvious changes were observed under low P. With RNA sequencing analysis, the authors confirmed that the phosphate starvation–induced (PSI) genes—including phosphate transporters *HvPHT1;1b*, *HvPHT1;1*, *HvPHT1;2*, and *HvPHO1;2*; transcription factor *HvMYB5Pc*; and major regulators *HvPHR1*, *HvPHR2*, and *HvPHR3*—were upregulated in *HvSPX4* knockout lines and repressed in *HvSPX4* overexpression lines (Zhang et al. 2025). This suggested that *HvSPX4* in barley functions as a negative regulator in low P response, similar to cases in rice and Arabidopsis.

As expected, HvSPX4 physically interacts with HvPHR1/2/4 transcription factors. This is experimentally shown with luciferase complementation imaging and yeast 2-hybrid assays (Zhang et al. 2025). In rice, this interaction is P dependent. OsSPX4 interacts and inhibits OsPHR2 function when P is sufficient, while OsSPX4 degrades through the ubiquitin/26S proteasome pathway under low P, thereby releasing OsPHR2 to activate downstream PSI genes (Lv et al. 2014). Similar P-dependent SPX4–PHR interactions and HvSPX4 degradation need to be investigated in barley.

Zhang et al. (2025) used 100 barley core genotypes for expression genome-wide association studies and separated them into low and high *HvSPX4* expression alleles based on their transcript levels (Fig. A and B). The high *HvSPX4* expression Allele 1 had a 50–base pair promoter region with 2 CAAT boxes, and it exhibited *HvSPX4* expression levels 1.5- to 2-fold higher than those of Allele 2. Notably, Allele 1 includes the low P–sensitive Salooni2, and the low P–tolerant Zaoaibai belongs to Allele 2.

**Figure.**
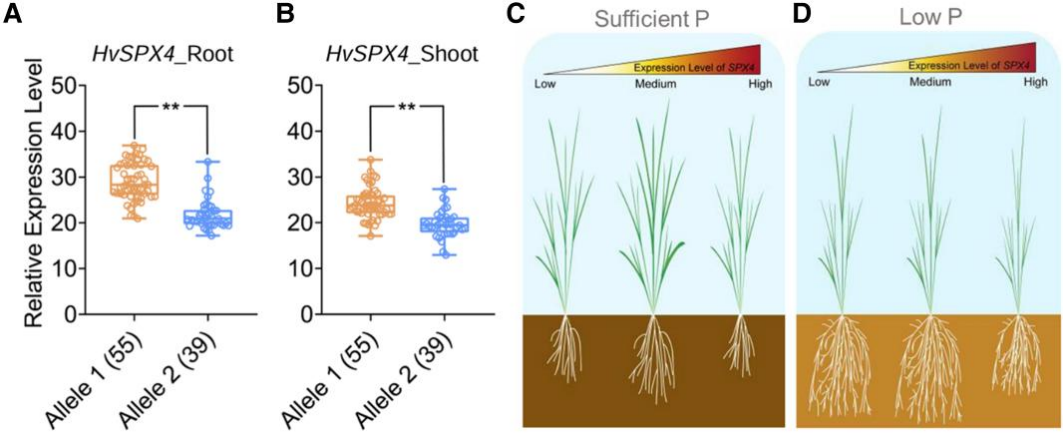
Expression levels of *HvSPX4* in barley accessions and a schematic presentation of *SPX4* expression levels affecting plant growth under low P. **A**, **B)**  *HvSPX4* expression in roots and shoots of barley accessions with 55 Allele 1 and 39 Allele 2. Data are presented as median (line), IQR (box), and 95% (error bars). **P < 0.01 (Student's *t* test). **C)**. Under sufficient P, a medium level of *SPX4* is essential for sustaining P homeostasis in plants, as either overabundance or deficiency of *SPX4* is harmful for plant growth. **D)** Under low P, a low or medium expression level of *SPX4* facilitates plant growth, whereas its overabundance reduces plant growth. The figure was modified from Zhang et al. (2025).

Consequently, the reduced *HvSPX4* expression in Allele 2 leads to increased expression of downstream PSI genes, such as *HvPHT1;6* and *HvPHO1;2*, under low P as compared with Allele 1. The Allele 2 genotypes also exhibit greater biomass in low P conditions (Zhang et al. 2025).

However, the *HvSPX4* overexpression lines with >15-fold increases in *HvSPX4* levels than in wild type, in turn, exhibited reduced barley growth, especially the root growth and architecture, under low and sufficient P supply (Zhang et al. 2025). Therefore, the expression level of *HvSPX4* appears to be critical for maintaining barley root growth as well. Under sufficient P, low and high expression levels of *SPX4* cause growth inhibition, whereas under low P, the low and medium expression levels of *SPX4* facilitate plant growth (Fig. C).

In brief, the research led by Zhang et al. (2025) highlights the critical function and fine-tuned expression level of *HvSPX4* for maintaining barley growth under low P. The finding provides a potentially valuable allele for developing low P–tolerant varieties.

## Data Availability

There are no data associated with this study.
